# Achieving Universal Health Coverage in Cambodia: Barriers, Strategies, and Policy Recommendations

**DOI:** 10.1002/hsr2.71259

**Published:** 2025-09-16

**Authors:** Virak Sorn

**Affiliations:** ^1^ Faculty of Health Sciences and Biotechnology University of Puthisastra Phnom Penh Cambodia

**Keywords:** Cambodia, challenges, health system, public health reform, Universal Health Coverage

## Abstract

**Background and Aim:**

Cambodia's Universal Health Coverage (UHC) score in 2021 was 58—lower than neighboring countries like Vietnam (68) and Thailand (82)—highlighting persistent challenges in healthcare access. Key issues include healthcare disparities, urban–rural inequalities, rising noncommunicable diseases (NCDs), and an overreliance on private healthcare services that increase out‐of‐pocket expenses. Therefore, this perspective article aims to discuss the challenges and strategies to address the issues of achieving UHC in Cambodia and the way forward.

**Methods:**

A literature review was conducted throughout May 2025 by retrieving articles from Google Scholar, PubMed, and gray literature from national and international organizations' websites, including the World Health Organization, United Nations, World Bank, and GIZ Cambodia. Additionally, I employed UHC, barriers, challenges, efforts, and Cambodia as the keywords.

**Results:**

Cambodia has made progress in healthcare access, including reductions in infant and maternal mortality and increased life expectancy. However, several systemic challenges hinder UHC advancement. These include significant healthcare quality and accessibility gaps between urban and rural areas, weak healthcare governance, and limited preventive measures for NCDs. The healthcare system is highly privatized, with only 20% of the population relying on public healthcare and 80% on private outpatient services—raising concerns about service quality and financial risk protection. Key barriers include socioeconomic inequality, inadequate infrastructure, and underdeveloped health financing mechanisms. To address these, strengthening the Health Equity Fund (HEF), improving infrastructure and human resources, investing in medicine storage, and establishing a “Cambodian Public Health Act” and “Public Health Commission” are recommended.

**Conclusions:**

Cambodia still has a long way to go to achieve UHC. Strengthening UHC will require policy reforms, strategies, and initiatives across a multisectoral approach. Cambodia's Public Health Commission could accelerate the country's journey toward UHC by utilizing best practices, information exchange, and collaborative approaches to maximize limited resources.

## 1 Introduction

Cambodia has made significant policy commitments in pursuit of Universal Health Coverage (UHC), including enacting national health strategies and aligning with global health development agendas. While these commitments have been in place for over a decade, progress toward achieving UHC remains uneven and slow [1]. Improved life expectancy and lower rates of infant and maternal mortality are just two of the notable improvements in population health indicators that Cambodia has achieved. In 2024, the overall fertility rate is 2.37 children per woman. While the infant mortality rate has significantly decreased from 78.1 to 18.67/1000 live births between 2000 and 2024, maternal mortality from 606 to 218/100,000 live births between 2000 and 2020, and life expectancy also improved from 58 to 70.74 years between 2000 and 2024 [2, 3, 4]. Yet, those achieved notable differences are noted based on area, caste, gender, class, and geography. Urbanization, socioeconomic disparity, and lack of access to high‐quality healthcare all present challenges to Cambodia's healthcare system [5]. Roughly 20% of Cambodia's total health market is served by public hospitals and HCs, which focus on the low‐income group. However, the private sector provides healthcare to the remaining 80% of the market through private clinics, hospitals, and pharmacies [6, 7]. On the other hand, almost 3/4 of the population lives in both rural and urban regions; the majority of healthcare professionals are concentrated in cities, with 2/4 of doctors and 3/4 of specialists employed in Phnom Penh, the nation's capital [6]. In light of these problems, authorities should concentrate on two main objectives: improving the quality of healthcare provided and expanding access to it for all Cambodians [7].

Cambodia's UHC service index score has improved in the last decade, but it still ranks poorly on effective UHC coverage with a score of 58 in 2021, compared to its neighbors like Vietnam (68), Thailand (82), and emerging economy peers like Singapore (89), Korea (89), Japan (83), and China (81), with the overall score of East Asia & Pacific being 75 [8]. In particular, there is still a lack of financial risk protection, with households paying about 55% in 2021 which increased to 61.21% in 2022 of all medical costs out‐of‐pocket; socioeconomic and geographic disparities still exist, and the quality of care and promoting health is unsettlingly low [9]. This results in a lack of access to effective healthcare services, diagnosis, treatment, and health check‐ups, which causes severe conditions and lowers the quality of life among the population and leads to an increase in the prevalence of both communicable diseases (CDs) and noncommunicable diseases (NCDs) in the country [10, 11]. Even though previous reports have recognized these issues, there is a lack of comprehensive analysis that critically examines how structural problems—like government gaps, private sector dynamics, urban–rural workforce disparity, and health financing—collectively impede Cambodia's progress toward UHC. Moreover, little has been written about feasible, forward‐looking strategies tailored to Cambodia's context. Therefore, this perspective article aims to discuss the challenges and strategies to address the issues of achieving UHC in Cambodia and the way forward.

## 2 Current Challenges

Cambodia's healthcare had improved step‐by‐step to provide better healthcare quality services to the people and achieve UHC. The country is working to expand coverage, but quality of care remains a significant challenge. The healthcare system is divided into two categories: public and private hospitals that use both for‐profit and nonprofit business plans. However, in real practice, outpatient curative consultations are typically provided by private sector facilities, while the majority of preventive services and inpatient care are provided by the public sector [12]. According to the Cambodian governance structure for healthcare facilities, there are three levels to the public healthcare system: the central, provincial, and operational districts. Every level of the health system has a specific role and function [7]. Currently, there are eight national hospitals, training facilities, national centers, and various Ministry of Health (MoH) departments at the central level. At the provincial level, which acts as a liaison between the operating district and central levels of the health system, there are 25 provincial health departments. There were 92 referral hospitals, 1222 health centers (HC), and 128 health posts at the operational district level, which is the most remote and population‐based subunit within the health system [13]. Although public healthcare was established for providing healthcare services to the population, the infrastructure was inadequate, and the availability of medical facilities, trained healthcare professionals, and essential medicines was limited, creating disparities between urban and rural healthcare services, which led to inconsistent or low‐quality healthcare services [6, 7, 8, 11]. This results in uneven service delivery and limited patient trust in public health facilities. Nonetheless, there has been a notable surge in the private sector lately, which now makes up 3/4 of primary care consultations in both urban and rural areas. The rise in private clinics has also resulted in an increase in out‐of‐pocket expenses, while certain private clinics are not effective in treating diseases that are somehow related to their services [7, 13]. This is related to the weakness of Cambodia's health governance, with limited oversight and enforcement of regulations, particularly in the private sector [7, 12]. Gaps in the supervision of healthcare accountability and standards are caused by the absence of a comprehensive legal framework.

On the other hand, there is a need for public health reforms, especially in healthcare promotion and preventive health. Because of the shift in dietary trends toward a higher consumption of sugar, salt, fat, and alcohol, there is a higher prevalence of NCDs in Cambodian society, including diabetes, cardiovascular diseases, and cancer. This presents new public health challenges for Cambodia and can result in addiction and mental health issues, which require long‐term care and management [6, 11]. Cultural beliefs, lack of awareness about health services, and the use of traditional medicine can deter people from seeking formal healthcare, particularly in rural areas [5, 6, 7, 12]. This is still a concern, which can lead to severe conditions. The other main issue is that government expenditure on health remains low, and the reliance on donor funding creates sustainability challenges since health financing needs to be more robust, with a focus on domestic resource mobilization to ensure long‐term sustainability [14, 15]. Although Cambodia has programs like the Health Equity Fund (HEF) and ID‐poor to assist the poor, out‐of‐pocket spending is still a significant issue. According to the report from Deutsche Gesellschaft für Internationale Zusammenarbeit (GIZ) GmbH, Cambodia in 2021, only 20% of the population as a whole might profit from the HEF project. The other program, the National Social Security Fund (NSSF) program, was established to safeguard and enhance the welfare of Cambodian workers. As of 2020, over 1.7 million people were enrolled with the NSSF within over 10,000 businesses that have been registered [7]. In 2024, about 7.5 million (42%) of the population was covered by both HEF and NSSF, according to the Royal Government of Cambodia's Roadmap Towards Universal Health Coverage in Cambodia 2024–2035 report, while NSSF has been used for health services at a higher rate than HEF since 2023 [9]. Even though this was highlighted as an achievement in healthcare improvement, it was a slight increase over a half decade compared to our neighbor countries like Vietnam, Thailand, and the other emerging economies in the region [8, 16, 17, 18].

Another issue is that the population of Cambodia has become less trusting of public healthcare due to its limited resources, treatment capacity, and effectiveness, all of which still require improvement [1, 4]. Since many Cambodians rely on private healthcare for outpatient care, which can be ineffective and expensive, it also leads to a serious condition. Informal payments to public healthcare workers are also common, increasing the financial burden on patients [6, 7]. To sum up, a number of factors, such as inadequate infrastructure, a shortage of healthcare professionals, restricted access to healthcare in rural areas, the quality of healthcare services, financial obstacles, a fragmented health system, health governance and regulation, public health reforms, public health financing, cultural and social barriers, and health information systems, made it difficult for Cambodia to achieve UHC [9, 10, 11, 12, 16, 17, 18, 19, 20, 21, 22]. To effectively address those issues, the government will have to put in a lot of effort, as indicated by the issue mentioned above.

## 3 Perspectives and Strategies

Sustainable Development Goal (SDG) 3 aims to attain UHC, which includes “financial risk protection, access to quality essential healthcare services, and access to safe, effective, quality, and affordable essential medicines and vaccines for all.” To achieve this, it requires a multisectoral approach. Strengthening the health system requires strong governance, leadership, and quality management infrastructure. External pressures and incentives can also stimulate performance improvement and quality in healthcare services [11, 16]. Based on expectations of sustained economic development, the government intends to raise spending, as outlined in the Health Strategic Plan 2016–2020, focused on two main tasks: the Health Information System Master Plan, aiming to address weaknesses in healthcare data quality, and the Health Workforce Development Plan, aiming to fill an expected shortfall in healthcare workers [4].

Recently, just 40% of the Cambodian population is now covered by social health protection, and 54% of people lack access to such coverage [1, 9]. The government will need to expand healthcare access in rural areas by investing in building and upgrading healthcare facilities, especially in rural and underserved areas, to improve access to essential services. Encouraging regular professional development training for healthcare providers is crucial to raising the standard of care [19, 20, 21]. Also, strengthen partnerships with universities and medical institutions to improve medical education and the skills of healthcare providers more effectively [21].

Despite obstacles like high out‐of‐pocket costs and a loosely regulated private sector, the Cambodian government is dedicated to enhancing healthcare access and quality. Strengthening the HEF and voucher schemes provides financial risk protection for the poor by expanding its amount to cover a larger proportion of the population, ensuring that financial barriers do not prevent access to healthcare [4, 23, 24]. The NSSF should be increasing its coverage capacity and reducing out‐of‐pocket payments. Initiatives like public–private partnerships could foster collaboration between the public and private healthcare sectors through clearly defined agreements [24, 25, 26]. Develop policies that allow the public sector to leverage the private sector's capacity while ensuring consistent care standards. Strengthen referral systems between primary, secondary, and tertiary healthcare levels to ensure smooth patient transitions between different healthcare facilities and improve the continuity of care [26, 27, 28]. However, promoting research on local health needs and outcomes to inform evidence‐based policies and interventions is a critical task to deal with, especially in collaboration with health universities or institutions.

Devolution of funds to district health societies and decentralization of planning, implementation, and monitoring can create a sustainable system rooted in provincial and local sociocultural contexts, empowering local health authorities to manage resources and services effectively [26, 27]. Broad policy frameworks, guidelines, and oversight, however, ought to stay at the federal level. Investing in health promotion and prevention, raising health literacy, and reducing costs associated with sickness care should be the primary healthcare teams' incentives. It is recommended that the government promote a comprehensive public health initiative that prioritizes health development and inclusive growth as societal goals [28, 29, 30]. Additionally, Cambodia needs to restructure its healthcare system, giving top priority to the adoption of the “Cambodian Public Health Act” to protect and promote the health of people as a fundamental right to health and healthcare and the creation of an autonomous “Public Health Commission” to carry out the act's mandate. The Public Health Commission should have enough resources (technical staff, equipment, office space, budget, and secretarial support staff) to enable the preparation, funding, implementation, and periodic monitoring of a Public Health Action Plan in collaboration with all relevant sectors to grant everyone the right to a standard and efficient health service to protect people from public health emergencies, improve UHC, and ensure better health and well‐being [6, 26, 29, 30, 31].

The enhancement of the health system has been found to depend critically on the provision of high‐quality care. Improving the way healthcare is delivered requires placing a high priority on the quality of services. Thus, strong governance and leadership are necessary to improve healthcare services and quality, particularly for the creation of a quality management infrastructure in both the public and private sectors. Additionally, outside incentives and pressures might promote quality and performance enhancement across the country [7]. Public health reform on healthcare policy decentralization and enhancing governance and regulation is needed. Addressing cultural and social barriers, reducing financial barriers, securing sustainable health financing, and promoting health and preventive care among Cambodia's population, especially in the urban and rural areas, will be impactful to Cambodia in achieving UHC as dreamed.

## 4 Policy Recommendations

To enhance readiness and successfully accomplish UHC in Cambodia, a planned, phased strategy that gives priority to both viability and long‐term sustainability is needed. The first priority should be strengthening primary health care (PHC) services, particularly in rural areas, by improving infrastructure, ensuring consistent availability of essential medicines, and increasing the number and capacity of community health workers. Second, private sector health providers must be strictly regulated and integrated into national health planning frameworks through licensing and accreditation standards and quality assurance to align with national public health goals. Third, Cambodia must implement a strong National Public Health Act to formally establish access, control service delivery, and uphold quality standards to institutionalize health as a right with mechanisms for accountability. Fourth, establishing an independent Public Health Commission can further ensure transparency, oversight, and evidence‐based decision‐making. Fifth, increasing domestic health financing through higher budget allocations and innovative mechanisms such as sin taxes (e.g., on tobacco and alcohol) can support the expansion of healthcare services [17]. Sixth, expanding and improving the NSSF and HEF is crucial to reducing financial barriers for the poor and informal sector workers, with efforts focused on enhancing targeting mechanisms and integrating HEF with broader social protection schemes. Seventh, a comprehensive health workforce strategy that prioritizes training and improves retention through salary incentives and rural hardship allowances will be necessary to address human resource challenges and maintain quality healthcare services delivery. Eighth, investing in digital health infrastructure, though a longer‐term goal, will be essential for enhancing healthcare data systems, service coordination, and improving access in remote areas [18, 26]. Though ambitious, these policy recommendations offer Cambodia a well‐organized roadmap for progressively achieving UHC, acknowledging that challenges such as limited resources, regulatory capacity, and political coordination must be systematically addressed.

## 5 Conclusion

The primary obstacles facing Cambodia in achieving UHC include inadequate health promotion, insufficient disease prevention efforts, limited medical supplies, a shortage of qualified healthcare staff at the PHC level, and an imbalanced healthcare delivery system. Reforms in public policy, along with multisectoral strategies and initiatives, will be necessary to address these challenges. A comprehensive multisectoral strategy must be designed and implemented to achieve SDGs and build public trust in health services nationwide. The establishment of a Public Health Commission, not only within the MoH but also across other relevant ministries and stakeholders, will significantly enhance the coordination of diverse programs aimed at improving healthcare accessibility and quality for all Cambodians. Clearly defined roles and strict regulations for both public and private healthcare providers will ensure high‐quality patient care while maintaining national and international standards. Finally, systemic changes, including improved governance, policy reforms, regulatory frameworks, and increased financial investment, are essential to sustaining progress in addressing Cambodia's healthcare challenges and moving the country closer to achieving UHC.

## Author Contributions

**Virak Sorn:** conceptualization, writing – original draft, writing – review and editing, resources.

## Ethics Statement

The author has nothing to report.

## Conflicts of Interest

The author declares no conflicts of interest.

## Transparency Statement

The author Virak Sorn affirms that this manuscript is an honest, accurate, and transparent account of the study being reported; that no important aspects of the study have been omitted; and that any discrepancies from the study as planned (and, if relevant, registered) have been explained.

## Data Availability

Due to the fact that no data sets were created or examined for this article, data sharing is not applicable.
